# Characterisation of equine odontoclastic tooth resorption and hypercementosis: A comparative study using microCT and radiography in age‐matched controls

**DOI:** 10.1111/evj.14453

**Published:** 2025-01-18

**Authors:** Zoe Nugent, Anders Jensen, Niamh Owen, Andrew J. Peffers, Mohesh Moothanchery, Mandy J. Peffers

**Affiliations:** ^1^ University of Liverpool, Institute of Life Course and Medical Sciences Liverpool UK; ^2^ North West Equine Dental Practice Ffrith UK; ^3^ Centre for Pre‐Clinical Imaging Liverpool UK

**Keywords:** ageing, EOTRH, horse, microCT, radiograph, teeth

## Abstract

**Background:**

Equine odontoclastic tooth resorption and hypercementosis (EOTRH) is a painful disorder primarily affecting the incisor teeth of horses over 15 years of age. Clinical signs of the disease include prehension problems, halitosis and in severe cases weight loss. The disease predominately affects the reserve crown and presents as a loss of dental tissue and excessive build‐up of cementum.

**Objectives:**

To determine the radiographic scores of horses with EOTRH and age‐matched controls and to increase understanding of EOTRH using microCT to compare teeth from horses with EOTRH and age‐matched controls.

**Study design:**

In vivo and ex vivo studies.

**Methods:**

This study used radiography (in vivo) and microcomputed tomography (microCT) (ex vivo) to help understand and help characterise the imaging changes that occur in EOTRH. For radiography, 87 patients were assessed using a radiographic scoring system for EOTRH. The microCT study was undertaken on 20 extracted cadaver incisor teeth which were scanned and segmented to measure the different dental tissues. These were assessed using a descriptive analysis (surface roughening, tooth resorption, root blunting and pulp cavity).

**Results:**

Radiographic scoring demonstrated that 03s were more severely affected than 01s in EOTRH. Total radiographic score and age had a weak positive correlation. Following microCT, we identified that EOTRH teeth had a lower pulp and enamel volume and therefore significantly higher pulp and enamel ratios relative to the whole tooth volume, compared with control teeth. Cementum and dentine volumes were more variable in EOTRH teeth. Thus, their ratios relative to the whole tooth volume were not different to control teeth.

**Main limitations:**

The number of horses was relatively small.

**Conclusions:**

Results suggest differing degrees of tooth resorption and hypercementosis, in different affected teeth potentially indicating multiple phenotypes of the disease. We provide further evidence of the presence of subclinical EOTRH imaging changes in some teeth.

## 1 INTRODUCTION

Staszyk et al.^1^ first described equine odontoclastic tooth resorption and hypercementosis (EOTRH) following its recognition clinically.^2^, ^3^, ^4^ It presents as destruction of calcified dental tissue and compensatory laying down of high levels of cementum, predominately along the intra‐alveolar part of the tooth.^1^, ^5^ EOTRH is predominately evident in the incisors,^6^ but it has been identified in canines and rarely in cheek teeth.^7^, ^8^ There is a notable increase in disease incidence with age, being commonly observed in horses over 15 years of age.^6^ Furthermore, it has been suggested that there is a male predilection.^9^ Although painful,^1^ EOTRH usually only becomes clinically apparent at an advanced stage.^6^ Gross changes include gingivitis, gingival recession and ulceration, periodontitis and increased tooth mobility. Fractures may occur due to the destruction of calcifies tissues. Clinical signs include prehension problems, halitosis, behavioural changes and in severe cases weight loss.^1^, ^5^, ^10^, ^11^

Currently, the only treatment option is removal of the affected teeth.^11^ Extraction is recommended when clinical examination reveals advanced changes, regardless of the presence of clinical signs. This is because most horse owners are often unaware of the disease's severity, and affected horses may continue to eat and perform normally despite the condition. Extraction is advised when the horse shows clinical signs, generally coinciding with when teeth are loose and severely resorbed below the gumline. However, in severe cases, patients require the extraction of all their incisors.^12^ Even though incisors are designed for the prehension of food material, horses can adapt by using their lips and tongue following extraction.^13^ Subsequent to full incisor extraction of EOTRH‐affected teeth the number of horses with a body condition score below a desired level was reduced by 50% and clinical signs were significantly reduced.^12^

The cause of EOTRH is unknown although some theories have been suggested,^14^ including strain to the periodontal ligament resulting in periodontitis^1^, ^15^ and red complex gram‐negative bacteria which have been isolated from cases of EOTRH.^16^ It is likely multifactorial but further research in larger populations and relevant controls is required to fully elucidate a potential cause.^14^

Although research into EOTRH is relatively limited, the majority of it has focused on diagnostic imaging through the use of radiography, microcomputed tomography (microCT) and computed tomography (CT).^6^, ^8^, ^11^, ^17^, ^18^, ^19^ When comparing microCT and radiography, the former is more sensitive. On the same sample of incisors radiography classified 10.2% as affected by EOTRH whereas microCT classified 69.3%.^17^ Whilst this illustrates that early stages of EOTRH may be missed by radiography, it is still a valuable tool in its diagnosis for the identification of horses without clinical signs.^20^ Additionally, it is a more practical method to undertake in vivo as radiographs can be taken using standing sedation.^19^ Although CT may be undertaken with standing sedation it is a more expensive option.^8^ For microCT investigations of teeth, samples need to be taken ex vivo.^17^

Previous radiographic research classified the severity of lesions by scoring teeth based on the system of Hüls et al.^21^ and adapted by Rehrl et al.^6^, ^19^, ^22^, ^23^ This scoring system categorised teeth on tooth resorption and hypercementosis with a total score out of three.^6^, ^21^ Albers et al.^17^ devised a different scoring system which recorded resorption and hypercementosis in more detail, including the assessment of tooth shape, structure and surface to give a score out of nine.^17^ Previous radiographic studies did not include the use of non‐EOTRH controls.^6^, ^11^, ^19^, ^22^ Indeed, little is understood regarding age‐related changes^14^ and so this study utilised age‐matched control horses.

The classification and stratification of EOTRH is important for early detection, personalised therapeutics and to increase our understanding of the disease. For radiographic analysis, we hypothesise that EOTRH patients would have a higher score compared with controls and that older horses would have higher scores. We also hypothesised that using microCT to compare teeth from horses with EOTRH and age‐matched controls would contribute to a deeper understanding of the disease's pathology and progression. This is because the technique enables a more precise, detailed and quantitative potentially leading to new insights into the disease's development, progression and underlying mechanisms.

## 2 MATERIALS AND METHODS

### 2.1 Radiography

**FIGURE 1:**
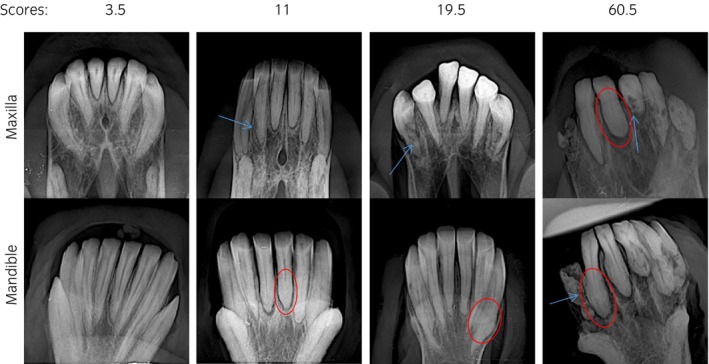
Representative radiographs showing the progression of EOTRH in four representative patients. Top row shows the maxillary teeth and the bottom row shows the mandibular teeth. Their score was devised from the adjusted radiographic scoring system of Albers et al.^17^ Blue arrows indicate tooth resorption and red circles indicate hypercementosis.

The radiographic data included 36 horses without an EOTRH diagnosis (control) patients and 51 EOTRH patients. This was a convenience population (a group of subjects selected for their availability and ease of access, rather than being randomly chosen to represent a larger population). These represented a mixture of sexes, ages and breeds (Table S1). Radiographs were taken at an Equine Dental Practice between October 2019 and March 2024 using a CDSR wireless equine DR (Vieworks, South Korea), using AJEX9020H generator. Maxillary incisors were viewed using a dorsoventral oblique view and mandibular incisors were viewed using a ventrodorsal oblique view. This was because it was the standard approach used in this dental practice, previously utilised in other studies and we wanted consistency throughout imaging. Radiographs that did not include both the maxillary and mandibular arcade of incisors and had missing teeth previously extracted were excluded. The inclusion criteria for non‐EOTRH patients were over 15 years of age and no history or clinical signs of EOTRH. The incisor teeth of each patient were scored using a modified scoring system of Albers et al.,^17^ due to the more detailed nature of this scoring system. An individual tooth could have a score of up to nine (Table 1). Patients were scored three times each and an average of these scores was obtained. Examples of radiographs taken for four patients and their scores are shown in Figure 1.

**TABLE 1:** Adjusted radiographical scoring system.^17^

Parameter	Characteristics	Score
Tooth shape	Normal	0
	Mild (blunted root tip or small enlargement in the occlusal half of the root)	1
	Moderate (blunted root tip and shortened tooth, but intra‐alveolar part of the tooth always narrower than the clinical crown)	2
	Severe (intra‐alveolar part of the tooth wider than the clinical crown)	3
Tooth surface	Regular/physiological	0
	Mild (single irregularity [<1/3 of the length of the intra‐alveolar part of the tooth])	1
	Moderate (1–2 irregularities or tooth appears as a whole slightly rough)	2
	Severe (more irregularities) or fracture	3
Tooth structure	Regular/physiological	0
	Mild (single radiolucency [<1/3 of the width of the intra‐alveolar part of the tooth])	1
	Moderate (several small radiolucencies [<1/3 of the width of the intra‐alveolar part of the tooth] or two radiolucencies [<2/3 of the width of the intra‐alveolar part of the tooth])	2
	Severe (more radiolucencies) or fracture	3

### 2.2 Microcomputed tomography

Teeth were extracted from a population of mixed sexes, ages and breeds (Table S2). The microCT data included 10 from horses without an EOTRH diagnosis (control) and 10 from horses with EOTRH teeth, including nine incisors and one canine. Teeth were extracted from EOTRH patients. This was a convenience population. Control teeth were collected from cadavers at an abattoir. Teeth were processed using a microCT system (Perkin Elmer, Quantum GX2). Teeth were scanned at high resolution with a scan time of 14 min. Scans used a copper and aluminium filter (Cu 0.06 mm + Al 0.5 mm), a voltage of 90 kV and a current of 88 μA. The field of view (FOV) was in the range of 72–86 mm to give an isotropic voxel size in the range of 144–172 μm.

DICOM files were exported and visualised using the software ITK‐SNAP. Automatic segmentation was undertaken. When this was not possible segmentation was undertaken manually. For each tooth the following were segmented: whole tooth (WT), pulp cavity (P) and enamel (E) to obtain volumes (mm^3^). As cementum and dentine (CD) were unable to be conclusively differentiated with microCT, these two tissues were measured and analysed together. To obtain the CD volume, P and E volumes were subtracted from the WT volume. Tooth resorption was subtracted from the overall volume of each tissue (WT‐R). Due to the hypsodont nature of equine teeth and their shortening as the horse ages, volume ratios of the P, E and CD were taken relative to the whole tooth volume. Descriptive analysis was used for the tooth which included surface roughening, tooth resorption, tooth shape and the pulp cavity.

### 2.3 Data analysis

Statistical analysis was performed using R programming version 4.2.2. The total radiographic score of teeth and ages of patients were tested for normality using a Shapiro–Wilk's normality test. Total score was non‐parametric so was compared using a Kruskal–Wallis test and pairwise comparisons were conducted using Dunn's test. Scores between control and EOTRH groups were analysed using a Mann–Whitney *U* test, and score and age were analysed using a *t*‐test. A Spearman's rank correlation was performed for age and sex correlation. Maxillary and mandibular scores were analysed using a t‐test and differences between incisor teeth 01s, 02s and 03s were looked at using a Dunn's test.

Ages of the teeth used for microCT, WT to P ratio, WT to E ratio and WT to CD were tested for normality using a Shapiro–Wilks normality test. Age of teeth was normally distributed so further analysis was conducted with a *t*‐test. For healthy and EOTRH, WT to P ratio and WT to E ratio were non‐parametric so analysed using Wilcox's test and Spearman's correlation. WT to CD ratio was normally distributed for healthy and EOTRH so were analysed using a *t*‐test and a Pearson's correlation. To assess the variability within and between the two groups a principal component analysis (PCA) was undertaken. The canine was not included in data analysis.

## 3 RESULTS

### 3.1 Radiography

**FIGURE 2:**
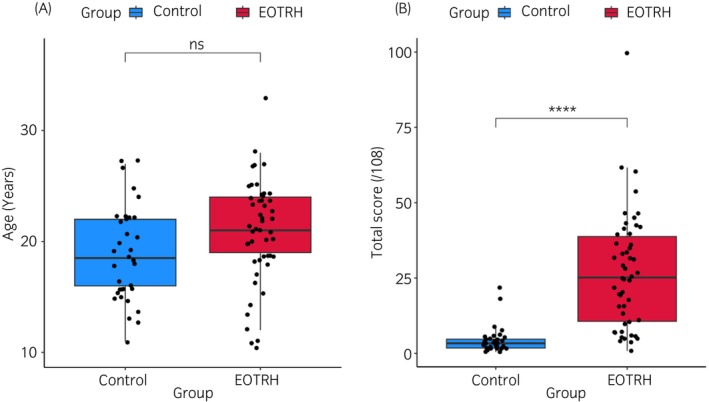
Graphs of age and radiographic scores between control and EOTRH groups. (A) Age. (B) Radiographic score. Blue represents control and red EOTRH. Statistical analysis is undertaken using a Mann–Whitney *U* test in R (**** *p* < 0.001).

There was no significant difference in the age between the control group, age range of 11–27 years (mean, 18.85 [years]) and EOTRH group, age range of 10–33 (mean, 20.86) (Figure 2A). There was a significant difference in radiographic score between the control group and the EOTRH group (*p* < 0.001) (Figure 2B). The scores undertaken by a single scorer (three independent occasions) were similar (Figure S1). Intraclass correlation coefficient had a single raters absolute of 0.96 and was significant (*p* < 0.001).

**FIGURE 3:**
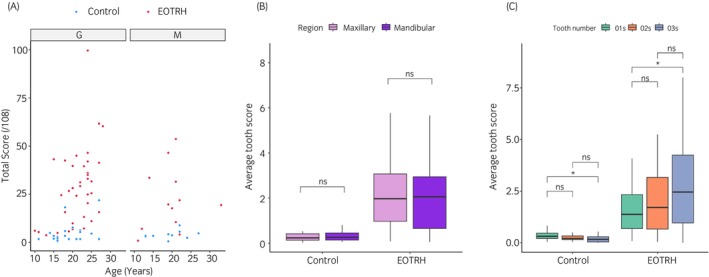
Radiographic scoring. (A) Spearman's rank correlation of age and total score in geldings and mares. Geldings (G) showed a weak correlation (cor = 0.51, *p* < 0.001), and mares (M) did not show a correlation between age and score (cor 0.24, *p* = 0.3). Control horses are in blue and EOTRH in red. (B) Comparison of maxillary and mandibular average teeth scores in control and EOTRH groups *t*‐test, NS; not significant. Maxillary teeth are represented in pink and mandibular in purple. (C) Comparison of 01s, 02s and 03s scores in control and EOTRH groups. Radiographic scores were lower in the 03s compared with the 01s in the control group and higher in the 03s compared with the 01s in EOTRH group (*p < 0.05, NS; not significant). Statistics is undertaken using a Dunns test in R.

There was no correlation between total score and sex in mares but a weak correlation between score and sex in geldings (*p* < 0.001). There was no significant difference between the ages neither of mares and geldings nor between total scores and sex. When ages and total score were compared without separating mares and geldings, there was a weak correlation; cor = 0.44, *p* < 0.001 (Figure 3A).

Average maxillary and mandibular tooth scores were not significantly different in both control and EOTRH groups (Figure 3B). When comparing scores between the 01s, 02s and 03s, differences were found between 01s and 03s for both control (*p* < 0.05) and EOTRH groups (*p* < 0.05). In the control group 03s scored lower than 01s whereas in the EOTRH group 03s scored higher than 01s (Figure 3C).

### 3.2 Microcomputed tomography

**FIGURE 4:**
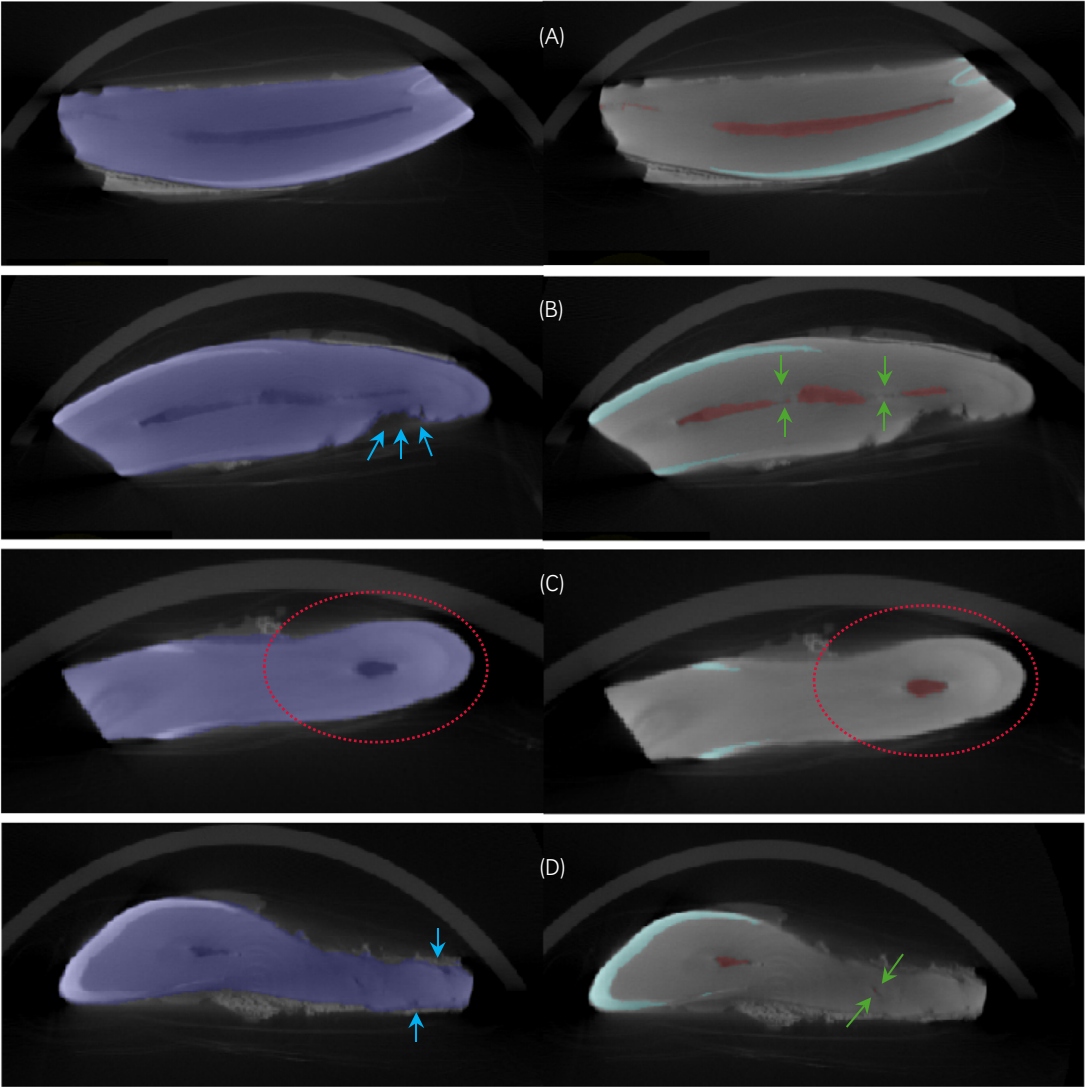
Microcomputed tomography images of control group. Axial view of teeth segmented by whole tooth (left), enamel and pulp cavity (right) of (A) H4 which shows no surface roughening or tooth resorption, a tapering root and normal pulp cavity, (B) H8 which shows moderate surface roughening (blue arrows) and tooth resorption and some mild resorption of the pulp cavity (green arrows), (C) H9 which shows mild blunting of the root (red dotted circle) and (D) H10 which shows very mild to mild surface roughening and tooth resorption and a moderately closed pulp cavity.

**FIGURE 5:**
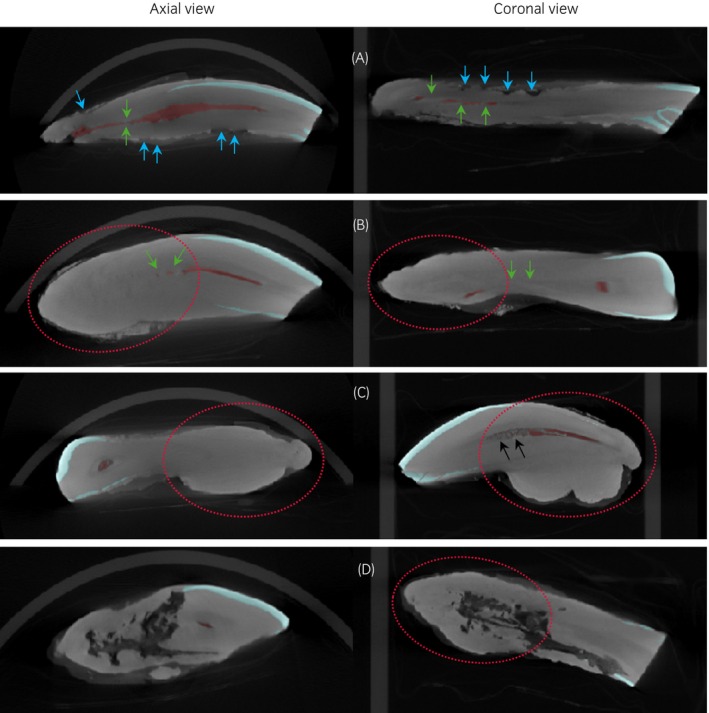
Microcomputed tomography (microCT) images of EOTRH group. Axial (left‐hand side) and coronal (right‐hand side) views of teeth segmented by enamel and pulp cavity of (A) D1 which shows moderate surface roughening (blue arrows), mild tooth resorption and a moderately closed pulp cavity (green arrows); (B) D3 which shows very mild surface roughening and tooth resorption, moderate root blunting (red dotted circle) and a severely closed pulp cavity; (C) D8 which shows a severely blunted root and severely closed pulp cavity with radiopaque material within (black arrows) and (D) D9 which shows severe tooth resorption, a mildly blunted root and a severely closed pulp cavity.

**FIGURE 6:**
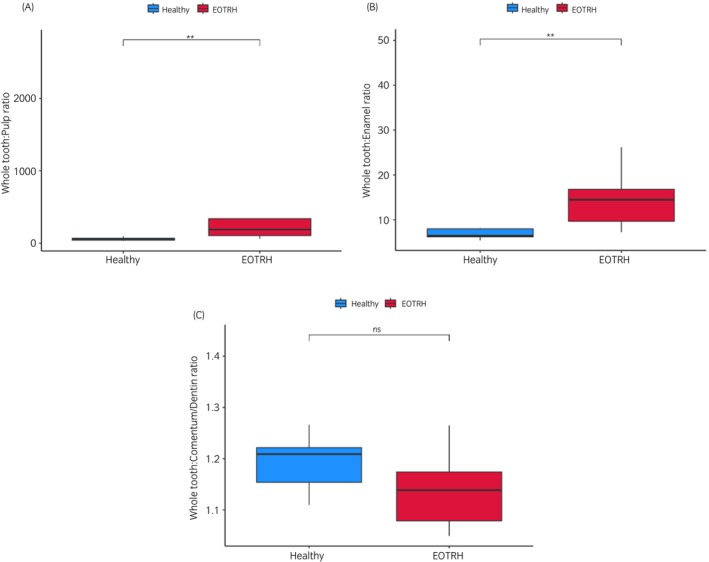
MicroCT measurement ratio in control and EOTRH. (A) Differences between whole tooth (WT) to pulp (P), (B) WT to enamel (E) and (C) WT to cementum/dentine (CD) ratios in control and EOTRH groups. Data are shown for control (blue) and EOTRH (red) groups. Wilcox's test used in R, **p < 0.01, ns; not significant.

There was no difference in age between the control group; age range of 17–22 (mean, 19.6 years) and the EOTRH group; age range of 18–25 (mean, 21.2 years). Figure 4 shows microCT images of the control group and Figure 5 shows microCT of the EOTRH group. Interestingly, some teeth from the control group showed some characteristics of EOTRH (Figure 6).

Whole tooth (WT) to pulp cavity (P) ratio was significantly increased in the EOTRH group (*p* < 0.01) (Figure 6A). WT to enamel (E) ratio volume was significantly increased (Figure 6B). As teeth from the EOTRH group showed enamel loss in the form of tooth resorption, this ratio was significantly higher (*p* < 0.01). The difference in WT to cementum and dentine (CD) ratio between control and EOTRH groups was not significant (Figure 6C). In teeth from the EOTRH group, there was greater variation in volume of CD (Table S3). There were variations between teeth in surface roughening and tooth resorption, and tooth shape indicative of hypercementosis. Figure 6C shows a wider range of WT:CD values for EOTRH teeth, but the findings did not reach significance.

**FIGURE 7:**
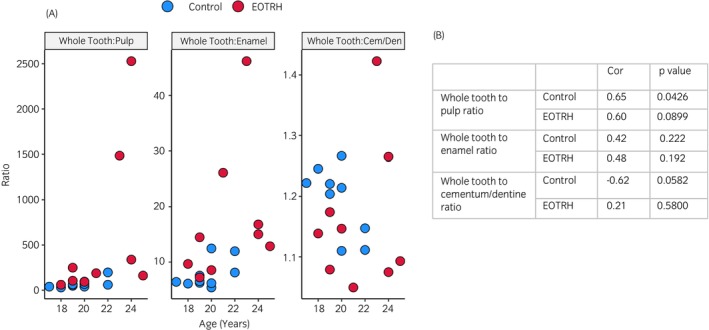
Correlation of teeth tissue with age. (A) Comparisons between whole tooth (WT) to pulp (P), WT to enamel (E) and WT to cementum/dentine (CD) ratios in control (blue) and EOTRH (red) groups. There was a significant correlation for WT:P in the control group (cor; 0.65, *p* < 0.05). (B) Table of correlation coefficient (cor) values and *p* values. Statistics using Pearson and Spearman's rank in R.

Only WT:P ratio in the control group showed a significant correlation with age (Figure 7). However, from the scatterplots for WT:P and WT:E ratios the control groups showed more uniformity whereas the EOTRH groups showed more variation. Similarly, the WT:CD ratio was more variable in its distribution for both control and EOTRH groups.

**FIGURE 8:**
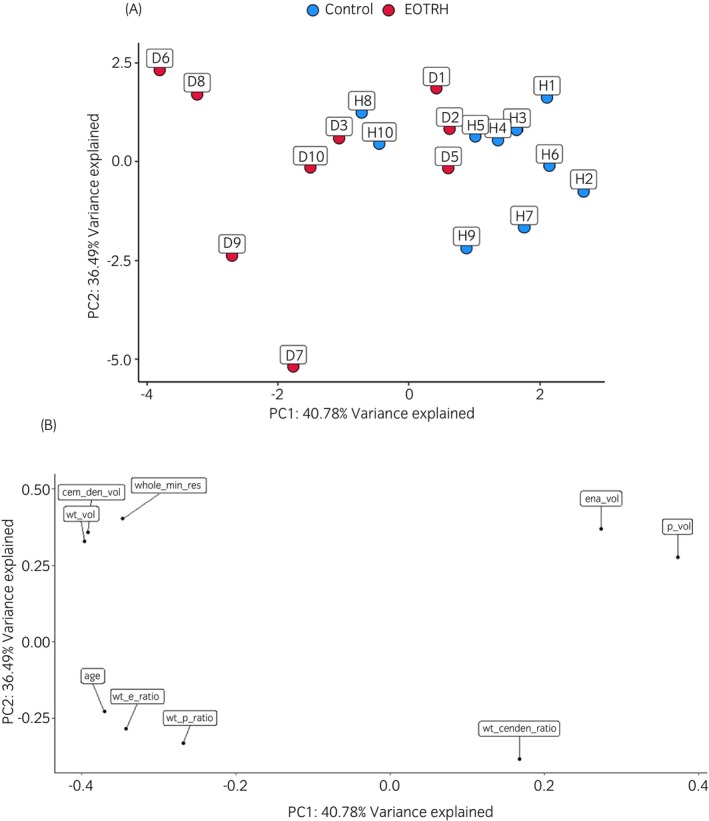
Plots exploring variation in microCT tooth tissue ratios between donors. (A) PCA plot, (B) loading plot to assess the variance between control (blue) and EOTRH (red) groups. PCA showed that there was more clustering of the control group and more variation within the EOTRH group. The loading plot visualised the relative weightings of each variable compared with PC1 and PC2. Image made in R.

As the EOTRH group showed more variability a PCA was conducted to look at variation within and between the two groups. Figure 8 shows that control samples were clustered together. However, H8, H9 and H10 were outside the cluster. These teeth showed some features consistent with EOTRH (Figure 7). H8 showed moderate surface roughening and mild hypercementosis (Table S3), thus a higher WT:P and WT:E ratio compared with the rest of the group. H9 showed some mild blunting (hypercementosis) of the root and H10 had some very mild tooth resorption with its pulp cavity moderately closed. This was reflected in H10's WT:P ratio which was high compared with the rest of the control group (Table S3). D1, D2 and D5 were more similar to the control cohort (Figure 8A). The descriptive analyses described that for the most parts they had mild surface roughening and tooth resorption, no hypercementosis and their pulp cavities were not severely closed (Table S3). Also, the WT:P and WT:E ratios for these three samples were the lowest in the EOTRH group. The loading plot showed that E and P volumes have a positive loading on principal component 1 (PC1) whereas WT:E and WT:P ratios have a negative loading on PC1 (Figure 8B).

## 4 DISCUSSION

EOTRH is an increasingly recognised equine dental disease, but little is known about its aetiology and progression. Previous radiographic,^6^, ^11^, ^18^, ^19^ computed tomography (CT),^8^ microcomputed tomography (microCT) and combinations of these imaging modalities^17^ studies have been undertaken. In our study we used aged, matched controls as previous studies lacked a comparison to age‐matched control horses.^6^, ^11^, ^19^, ^22^ We utilised radiography (in vivo) and microCT (ex vivo) with microCT being more sensitive than radiography.^17^ To our knowledge this is the first study to undertake radiographic and microCt studies using a UK population.

Our findings indicated that differences between control and EOTRH teeth were not solely due to age. EOTRH is a disease which increase in prevalence with age^1^ but its aetiology is considered multifactorial.^15^, ^16^ Our findings indicate that EOTRH is not solely a consequence of increased age, as there was no significant difference in age between the control group and the EOTRH group in the radiographic study. Although there was a positive correlation between age and score in geldings, this was not evident in mares. However, when score and age were correlated without sex as a factor, there was a positive correlation between score and age. This positive correlation in geldings may, however, be linked to sample size. There was no difference in total score between mares and geldings which is contrary to previous research.^9^, ^11^ However, there were fewer mares than geldings in the dataset. This highlights that further research is required to determine whether there are any sex predilections.

The 03s showed a higher score than the 01s in the EOTRH group. This concurs with previous findings,^5^, ^9^, ^11^ suggesting that EOTRH does not affect the oldest tooth first, as the 01s are the oldest incisors and 03s the youngest^24^ Strain to the periodontal ligament has been considered as a factor contributing to EOTRH.^15^ A 3D model demonstrated that older horses had larger areas of higher stress on the periodontal ligament compared with younger horses.^15^ Additionally, these areas were similar to where initial lesions of EOTRH have been shown to progress from histologically.^15^ The 01s and 02s are surrounded by adjacent teeth unlike the 03s and it has been hypothesised this may contribute to the aetiology of EOTRH.^9^ However, Schrock et al.^15^ did not document differences in the periodontal ligament between 01s, 02s and 03s so it is not possible to elucidate whether different teeth have different periodontal ligament forces which may predispose to EOTRH.

Schrock et al.^25^ demonstrated that maxillary 02s were longer than maxillary 01s and 03s, which are similar in length. Mandibular 03s were longer than 01s.^25^ Whilst undertaken on a population of various breeds of horses, this study illustrated that teeth had different lengths.^25^ Longer teeth have more periodontal ligament whereas shorter teeth have less, and this may be a factor in predisposition to EOTRH. Results in the control group demonstrated that the 01s had a higher score than the 03s. Whilst this suggests older teeth (01s) were affected by changes before younger teeth (03s), it could be due to the radiographic views making visualisation of the 03s more difficult. The dorsoventral oblique views used favoured visualisation of the 02s and 01s, resulting in any subtle changes being more likely to be evident. This could contribute to them having a higher score overall. To limit this bias, additional radiographic images should be taken favouring views of the 03s enabling mild changes to be more reliably documented. Indeed, it should be emphasised that a single view may not capture the full extent of the disease, potentially leading to underdiagnosis or misinterpretation of the severity of EOTRH. This is a crucial consideration for veterinary practitioners aiming for accurate diagnosis.

Rehrl et al.^6^ found that horses over 14 years of age without clinical evidence of EOTRH displayed at least mild changes consistent with the disease. In our control group, some horses exhibited subtle changes consistent with EOTRH, suggesting some radiographic signs could be age‐related rather than EOTRH‐related changes. However, our control group was relatively small compared with the EOTRH group and a larger study is required to confirm this. Furthermore, the control group included two outliers which had a high score. This implies some horses with EOTRH are not being diagnosed early enough, aligning with previous work.^20^, ^26^ This highlights the importance of radiography for the diagnosis of EOTRH in horses without clinical signs.

The breeds of most horses in this study were unknown. Therefore, it was not possible to draw any conclusions about breed predilection. Future studies could include this metadata. Furthermore, data on horses' management including what and how they were fed could be included to identify if radiographic scores correlate with any management factors in the incidence of EOTRH. This was undertaken in previous studies.^23^ Masey O'Neill et al.^27^ found that horses fed on a higher concentrate diet had an increased incidence of dental disease compared with those that lived at pasture thus fed a more fibre‐based diet. Whilst this study was describing dental disease more widely, these could be risk factors in EOTRH.

As there was no difference in ages of horses between the control and EOTRH groups, differences were probably not age‐related. WT:P ratio was increased in the EOTRH group. This ratio corresponded with the descriptive analysis of the teeth as EOTRH teeth were more likely to have a reduced pulp cavity due to the disease process. Therefore, there was reduced pulp volume increasing the WT:P ratio. Similarly, WT:E ratio was increased in the EOTRH group. This was due to resorption causing a loss of enamel thus increasing the ratio. This finding was also consistent with the descriptive analysis of the teeth; the more surface roughening and tooth resorption the more likely the WT:E ratio was increased. WT:CD ratio was not significantly different between the control and EOTRH groups. This illustrated that EOTRH teeth were much more varied in their CD volumes. WT:CD ratios were higher in teeth predominantly displaying tooth resorption as there was loss of cementum and dentine. However, the ratio was lower in teeth predominantly displaying hypercementosis in which there was the addition of cementum. In teeth, with a mixture of both signs there was little change in the ratio between the control and EOTRH groups. This highlighted the importance of using descriptive analysis alongside quantitative analysis. Although most of the WT:P, WT:E and WT:CD ratios showed no correlation with age it was clear that the control cohort were more uniform. Teeth from horses with EOTRH had a greater variability in their ratios and these were more likely to be higher. This was supported by PCA analysis as teeth from the control group were clustered together. There were three teeth outside the control cluster and these displayed characteristics more consistent with EOTRH. These three outliers were the oldest in the control cohort which aligns with EOTRH as a disease that increases with age.

There were three EOTRH teeth with fewer characteristics of EOTRH which explained their similarly to the control cohort on the PCA plot. These three teeth were amongst the youngest in the EOTRH group. Variables close to each other on the loading plot were positively correlated. The loading plot demonstrated that pulp and enamel volume were correlated with each other as they both had a positive loading on principal component 1 (PC1). This link can be explained as higher pulp and enamel volumes were likely to be a finding in control teeth and low pulp and enamel volumes were likely to be a finding in EOTRH teeth. Similarly, WT:E and WT:P ratios were correlated with each other as they both negatively correlated with PC1. This was consistent with control teeth with a lower WT:P and WT:E ratio and EOTRH teeth with a higher WT:E and WT:P ratio.

Bearth et al.^8^ found that horses with EOTRH had a reduced pulp cavity length and length of enamel of the palatal/lingual or labial side of the tooth. These findings correspond to our results as EOTRH teeth had lower pulp cavity and enamel volumes reflected in their higher ratios relative to the whole tooth. Some pulp cavities of EORTH teeth contained radiolucent areas and this was particularly evident for D8. Interestingly using histology to study horses with EOTRH, atubular‐mineralised material was noted in the pulp cavity.^1^, ^9^ This material is classed histologically as cementum but resembles fibrodentine,^1^ an intermediate product that precedes tertiary dentine.^1^

Our study had several limitations. Using microCT alone we were unable to differentiate between cementum and dentine, so they were measured together. As EOTRH is a disease that affects cementum (as well as enamel and pulp), it would be more useful if there was a method to undertake measurements separately so that more accurate quantification and conclusions regarding this tissue could be made. Schrock et al.^25^ undertook microCT of maxillary and mandibular incisor arcades and for each sample 1000–2000 2D microCT images were taken. By maximising the noise‐to‐signal ratio, improved contrast of cementum and dentine was achieved.^25^ In contrast, our scans produced 270 images meaning differentiation between the tissues was not possible. Another approach could be to scan the teeth using magnetic resonance imaging (MRI) and combine it with microCT images as MRI can differentiate between cementum and dentine. Whilst horses are hypsodont and their incisors are used to estimate their age,^28^ this is only an estimate suggesting that incisors erupt at slightly different rates. Therefore, although we used ratios to relate back to the overall whole tooth volume, potential differences in the normal shortening may need to be considered. The incisors were extracted to be scanned ex vivo and although care was taken during extraction, it is possible some surface roughening could be due to extraction forces on the teeth. CT scanning of the incisor arcades may overcome this. However, it is impractical to carry out microCT in vivo. In addition, due to a lack of understanding regarding the normal ageing process of equine teeth, it is difficult to definitively attribute subtle EOTRH‐like changes to disease rather than age. Lastly, a combination of radiography (in vivo) and microCT (ex vivo) could be undertaken to examine their sensitivities relative to each other.

## 5 CONCLUSION

Our use of radiography (in vivo) and microCT (ex vivo) has contributed to increased understanding of the disease. Our groups were age‐matched thus changes evident were due to factors other than age. The scoring system used for radiographic images was repeatable. The radiographic scores were not affected by the tooth being in the maxilla or mandible. The 03s had a higher score compared with the 01s in the EOTRH group. There was a weak positive correlation between total score and age. EOTRH teeth had a lower pulp and enamel volume meaning their ratios relative to whole tooth volume were higher compared with control teeth. The cementum and dentine volumes were more variable in EOTRH depending on whether resorption or hypercementosis was dominant. Our study provides further evidence of the presence of subclinical EOTRH imaging changes in some teeth.

## FUNDING INFORMATION

Anders Jensen was funded by The Dunhill Medical Trust.

## CONFLICT OF INTEREST STATEMENT

The authors have declared no conflicting interests.

## AUTHOR CONTRIBUTIONS

**Zoe Nugent:** Conceptualization; investigation; writing – original draft; methodology; writing – review and editing; formal analysis; data curation. **Anders Jensen:** Investigation; writing – review and editing; formal analysis; supervision. **Niamh Owen:** Investigation; writing – review and editing; formal analysis. **Andrew J. Peffers:** Investigation; writing – review and editing; methodology; resources. **Mohesh Moothanchery:** Methodology; data curation; supervision; writing – review and editing. **Mandy J. Peffers:** Conceptualization; investigation; funding acquisition; writing – original draft; writing – review and editing; formal analysis; supervision.

## DATA INTEGRITY STATEMENT

Mandy J Peffers had full access to all the data in the study and takes responsibility for the integrity of the data and the accuracy of the data analysis.

## ETHICAL ANIMAL RESEARCH

Ethical approval was through the University of Liverpool Ethics Committee (VREC 1424).

## INFORMED CONSENT

Data was used with informed consent by the owners.

### PEER REVIEW

The peer review history for this article is available at https://www.webofscience.com/api/gateway/wos/peer-review/10.1111/evj.14453.

## Supporting information


**Figure S1.** Graph of three radiographic scores undertaken.

**Table S1.** Table of sex, age and breeds of horses used in radiographic study.

**Table S2.** Age and sex of horses whose teeth were used in micro‐computed tomography study.

**Table S3.** Quantitative analysis from microcomputed tomography images of teeth.

## Data Availability

The data that support the findings of this study are openly available in Figshare at https://doi.org/10.6084/m9.figshare.28163417.v1.
