# Supplementary material for: Characterisation of equine odontoclastic tooth resorption and hypercementosis: A comparative study using microCT and radiography in age‐matched controls
Source: Equine Vet J. 2025 Jan 18;57(4):1099–109. doi: 10.1111/evj.14453 (PMC12135745; doi:10.1111/evj.14453)

Equine Veterinary Journal BEVA

Figure S1: Graph of three radiographic scores undertaken. Results following scoring by the same scorer. A Kruskal-Wallis followed by a Dunn's test was used an to look at differences between score 1, 2 and 3. Some scores are labelled on the graph, D = EOTRH, number indicates patient's ID.

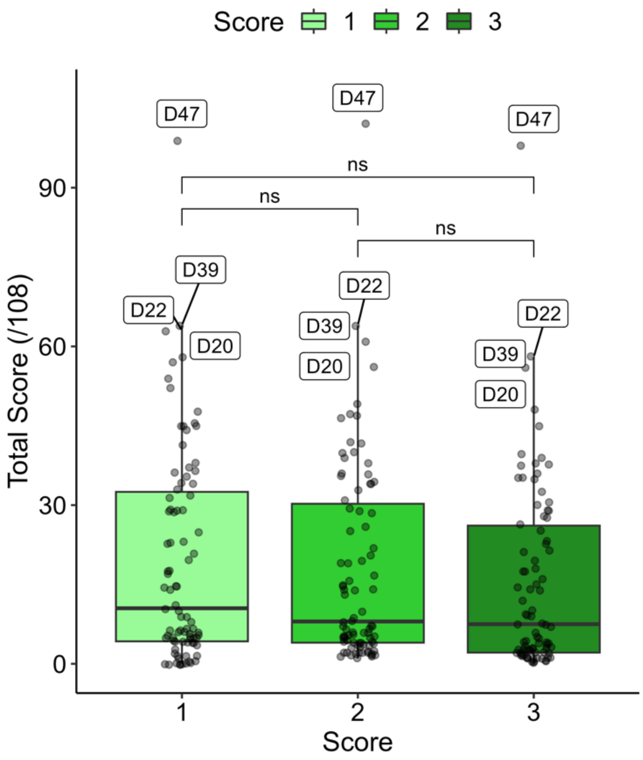

Supplement: Supplementary file 1 — Figure S1. Graph of three radiographic scores undertaken. [file EVJ-57-1099-s003.pdf]
