# Supplementary material for: Characterisation of equine odontoclastic tooth resorption and hypercementosis: A comparative study using microCT and radiography in age‐matched controls
Source: Equine Vet J. 2025 Jan 18;57(4):1099–109. doi: 10.1111/evj.14453 (PMC12135745; doi:10.1111/evj.14453)
Supplement: Supplementary file 2 — Table S1. Table of sex, age and breeds of horses used in radiographic study. [file EVJ-57-1099-s001.pdf]

Table S1: Table of sex, age and breeds of horses used in radiographic study.

ID	Sex	Age	Breed
D1	G	21	Dutch Warmblood
D2	G	19	Cob
D3	G	25	Dutch Warmblood
D4	M	19	
D6	G	24	Welsh Sec D
D7	G	21	
D8	M	33	
D9	M	20	Thoroughbred
D10	G	24	
D11	G	23	Irish Draught
D12	M	21	Irish Sports Horse
D13	G	20	
D14	M	21	Thoroughbred
D15	G	27	Welsh Cob
D16	G	23	
D17	M	19	
D18	G	24	
D19	M	14	Cross Breed
D20	M	21	
D21	G	24	Connemara
D22	G	27	
D23	G	22	Welsh Sec D
D24	G	13	
D25	M		
D26	M	24	
D27	G	21	Welsh Sec D
D28	G	15	
D29	G	25	

D30	M	22	
D31	G	10	Welsh Sec D
D32	G	22	Welsh Sec C
D33	M	12	Irish Sports Horse
D34	G	20	Irish Sports Horse
D35	M	11	Cob cross
D36	G	24	Thoroughbred
D37	G	17	
D38	M		
D39	G	28	
D40	G	25	
D41	G	18	
D42	G	11	Welsh Sec B
D43	G	23	
D44	M	22	
D45	G	16	Sports horse
D46	G	24	Anglo Arab cross Welsh Sec B
D47	G	24	
D48	G	19	
D49	G	24	
D50	G	27	Welsh Sec D
D51	G	19	
D52	G	18	Connemara
D53	G	18	Warmblood
D54	G	20	Thoroughbred
H1	G	16	Cob
H2	G	21	Welsh Sec D
H3	G	16	Arab cross
H4	G	11	
H5	G	22	Irish Sports Horse
H6	G	16	
H7	G	20	

H8	G	27	
H9	M	22	
H10	M	27	
H11	G	16	Comtois
H12	G	22	Cob
H13	M		
H14	G	15	Irish Sports Horse
H15	M		
H16	G	18	Thoroughbred
H17	M	13	
H18	G	16	
H19	G	18	
H20	G	15	Cob
H21	G	22	Cob
H22	M	22	
H23	G	25	Irish Sports Horse
H24	G	14	Arab cross
H25	M	20	Shetland
H26	M	24	Arab
H27	G	15	Arab
H28	M	15	
H29	G	18	Arab cross Welsh Cob
H30	S	16	Arab
H31	G	19	Cob
H32	M	19	
H33	G	22	
H34	G	27	Welsh Cob
H35	M	19	Irish Sports Horse
H36	M	13	

D = EOTRH, H = controls, G = gelding, M = mare
