# Supplementary material for: Characterisation of equine odontoclastic tooth resorption and hypercementosis: A comparative study using microCT and radiography in age‐matched controls
Source: Equine Vet J. 2025 Jan 18;57(4):1099–109. doi: 10.1111/evj.14453 (PMC12135745; doi:10.1111/evj.14453)
Supplement: Supplementary file 3 — Table S2. Age and sex of horses whose teeth were used in micro‐computed tomography study. [file EVJ-57-1099-s004.pdf]

Table S2: Age and sex of horses whose teeth were used in micro-computed tomography study.

**ID**	**Age**	**Sex**
H1	17	-
H2	18	Gelding
H3	19	Mare
H4	19	-
H5	19	-
H6	20	Mare
H7	20	Mare
H8	20	-
H9	22	Mare
H10	22	-
D1	18	Gelding
D2	19	Mare
D3	19	-
D4	19	-
D5	20	Gelding
D6	21	Gelding
D7	23	-
D8	24	Gelding
D9	24	Mare
D10	25	-

H = control, D = EOTRH.
