# Supplementary material for: Characterisation of equine odontoclastic tooth resorption and hypercementosis: A comparative study using microCT and radiography in age‐matched controls
Source: Equine Vet J. 2025 Jan 18;57(4):1099–109. doi: 10.1111/evj.14453 (PMC12135745; doi:10.1111/evj.14453)
Supplement: Supplementary file 4 — Table S3. Quantitative analysis from microcomputed tomography images of teeth. [file EVJ-57-1099-s002.pdf]

Equine Veterinary Journal BEVA

Table S3: Quantitative analysis from microcomputed tomography images of teeth.

Tooth ID	Age	Whole tooth (WT) volume (mm^3)	Pulp cavity (P) volume (mm^3)	WT:P	Enamel (E) volume (mm^3)	WT:E	Cementum and dentine (CD) volume (mm^3)	WT:CD	WT-R (resorption)
**H1**	17	5584	141.4	39.49	870	6.42	4569.6	1.22	5581
**H2**	18	3253	108.3	30.04	532.9	6.10	2611.8	1.25	N/A
**H3**	19	5103	104.8	48.69	817.1	6.25	4181.1	1.22	N/A
**H4**	19	4984	80.99	61.54	763	6.53	4141.01	1.20	N/A
**H5**	19	5184	80.08	64.74	686.8	7.55	4416.12	1.17	5183
**H6**	20	4172	105.4	39.58	771.8	5.41	3294.8	1.27	N/A
**H7**	20	2848	41.49	68.64	460.7	6.18	2345.81	1.21	N/A
**H8**	20	6674	69.71	95.74	535.7	12.46	6013.59	1.11	6619
**H9**	22	2495	41.36	60.32	208.6	11.96	2245.04	1.11	N/A
**H10**	22	5814	29.5	197.08	716.5	8.11	5068	1.15	N/A
**D1**	18	6565	108.5	60.51	679.6	9.66	5765.9	1.14	6554
**D2**	19	5592	53.41	104.70	774.4	7.22	4763.19	1.17	5591
**D3**	19	6194	24.86	249.16	427.9	14.48	5741.24	1.08	N/A
**D5**	20	4621	50.05	92.33	540.5	8.55	4030.45	1.15	N/A
**D6**	21	9523	50.61	188.16	364.4	26.13	9075.99	1.05	9491
**D7**	23	4370	2.95	1483.37	94.58	46.20	3071.474	1.42	3169
**D8**	24	8532	25.21	338.44	567.7	15.03	7939.09	1.07	N/A
**D9**	24	6588	2.61	2528.98	392.1	16.80	5208.295	1.26	5603
**D10**	25	5754	35.56	161.81	447.5	12.86	5264.94	1.09	5748

H = control, D = EOTRH, WT = whole tooth, P = pulp cavity, E = enamel, CD = cementum and dentine.
